# A Quantitative Risk Assessment Model for *Listeria monocytogenes* in Ready-to-Eat Cantaloupe

**DOI:** 10.3390/foods14132212

**Published:** 2025-06-23

**Authors:** Laurent Guillier, Ursula Gonzales-Barron, Régis Pouillot, Juliana De Oliveira Mota, Ana Allende, Jovana Kovacevic, Claudia Guldimann, Aamir Fazil, Hamzah Al-Qadiri, Qingli Dong, Akio Hasegawa, Vasco Cadavez, Moez Sanaa

**Affiliations:** 1 Risk Assessment Department, French Agency for Food, Environmental and Occupational Health & Safety (Anses), 14 rue Pierre et Marie Curie, 94700 Maisons-Alfort, France; 2 CIMO, LA SusTEC, Instituto Politécnico de Bragança, Campus de Santa Apolónia, 5300-253 Bragança, Portugal; ubarron@ipb.pt (U.G.-B.); vcadavez@ipb.pt (V.C.); 3 Consultant, 18 rue Mohamed Al Ghazi, Rabat 10170, Morocco; rpouillot.work@gmail.com; 4 Department of Nutrition and Food Safety, World Health Organization, 1211 Geneva, Switzerland; deju@who.int (J.D.O.M.); hasegawaa@who.int (A.H.); 5 CEBAS-CSIC, Microbiology and Quality of Fruits and Vegetables Group, Campus de Espinardo, 25, 30100 Murcia, Spain; aallende@cebas.csic.es; 6 Food Innovation Center, Oregon State University, 1207 NW Naito Parkway, Portland, OR 97209, USA; jovana.kovacevic@oregonstate.edu; 7 Competence Center for Food Safety, Chair for Food Safety and Analytics, Faculty of Veterinary Medicine, Ludwig-Maximilians-Universität München (LMU), Schönleutnerstrasse 8, 85764 Oberschleissheim, Germany; c.guldimann@lmu.de; 8 Public Health Risk Sciences Division (PHRS), Public Health Agency of Canada, 370 Speedvale Avenue West, Guelph, ON N1H 7M7, Canada; aamir.fazil@phac-aspc.gc.ca; 9 Department of Nutrition and Food Technology, School of Agriculture, The University of Jordan, Amman 11942, Jordan; h.qadiri@ju.edu.jo; 10 School of Health Science and Engineering, University of Shanghai for Science and Technology, Shanghai 200093, China; qdong@usst.edu.cn

**Keywords:** melon, primary production, listeriosis, exposure assessment, simulation

## Abstract

This study introduces a farm-to-fork quantitative risk assessment (QRA) model for invasive listeriosis from ready-to-eat diced cantaloupe. The modular model comprises seven stages—preharvest (soil and irrigation contamination), harvest (cross-contamination and survival), pre-processing (brushing), processing (flume tank washing, dicing and equipment cross-contamination), lot testing, cold-chain transport and retail growth, and consumer storage/handling. Each stage employs stochastic functions to simulate microbial prevalence and concentration changes (growth, inactivation, removal, partitioning, cross-contamination) using published data. In a reference scenario—good agricultural practices (soil barriers, no preharvest irrigation), hygienic processing and proper cold storage—the model predicts low lot- and pack-level contamination, with few packs >10 CFU/g and most servings below detection; the mean risk per serving is very low. “What-if” analyses highlight critical control points: the absence of soil barriers with preharvest irrigation can increase the risk by 10,000-fold; flume tank water contamination has a greater impact than harvest-stage cross-contamination; and poor consumer storage can raise the risk by up to 500-fold. This flexible QRA framework enables regulators and industry to evaluate and optimize interventions—from improved agricultural measures to targeted sampling plans and consumer guidance—to mitigate listeriosis risk from RTE diced cantaloupe.

## 1. Introduction

Historical data on foodborne outbreaks demonstrate that cantaloupes are frequently implicated in incidents of disease caused by foodborne pathogens, posing a substantial public health concern. Common implicated pathogens include Shiga toxin-producing *Escherichia coli* (STEC), *Salmonella* [1,2,3,4], norovirus [5], and *Listeria monocytogenes* [6,7]. Given that listeriosis cases mostly occur sporadically [8], findings from a meta-analysis of case–control studies focusing on sporadic listeriosis [9] highlight the role of the consumption of produce as a significant risk factor among various categories of ready-to-eat (RTE) foods. Notably, within the produce category, fruit consumption—specifically melons, cantaloupes, strawberries, and RTE fruit salads—was significantly associated with an increased risk of listeriosis among susceptible populations (pooled odds ratio = 1.538; 95% CI: 1.1431–2.070).

Given the ubiquitousness of *L. monocytogenes* in the environment, multiple routes of contamination for cantaloupes can be identified. *Listeria* species thrive in diverse habitats, predominantly in various soil types where they exist at low levels, below 10^4^ CFU·g^−1^ [10,11]. Additionally, *L. monocytogenes* colonizes the root systems of plants [12,13] and is hosted by various soil-dwelling organisms, including unicellular eukaryotes, nematodes, isopods, dipterans, and beetles [14,15]. Agricultural soils can further be contaminated through the application of organic fertilizers [16,17]. Beyond soil, irrigation water serves as another contamination route, with studies identifying it as a risk factor for *L. monocytogenes* presence in agricultural fields [16,18]. The inherent growth pattern of cantaloupes, in direct contact with the ground, further increases their vulnerability to contamination from soil and irrigation water. Additionally, the intricate net-like structure of the rind provides an ideal surface for pathogenic bacteria to adhere and persist on the fruit’s surface [19,20].

An additional route of contamination is throughout the different processing steps after harvesting. Despite the implementation of good hygiene practices and environmental controls in production settings, it has been observed that some strains of *L. monocytogenes* can establish themselves within (any) food processing environment and remain there for several months, or even years [21,22]. The investigation of the 2011 outbreak linked to cantaloupes that resulted in 147 listeriosis cases in the United States (US) allowed identifying several potential factors that may have contributed to the contamination. These factors were particularly associated with the food contact surfaces in a cantaloupe packing facility [23]. In 2013, 17 cantaloupe packinghouses in US were surveyed by the FDA, examining both food-contact and non-food-contact surfaces for *L. monocytogenes*. The survey reported *L. monocytogenes* at one facility and nonpathogenic *Listeria* species at eight others [24]. A research study involving Florida cantaloupe packinghouses, which investigated the prevalence of *L. monocytogenes* and indicator microorganisms, found a low prevalence of *L. monocytogenes* in 2013 (2/374 samples), and no detected *L. monocytogenes* in subsequent testing in 2014 [25]. Notably, total plate counts, coliforms and *E. coli* were found to be poor indicators of *L. monocytogenes* presence in cantaloupe packinghouse environments [25]. The role of water use during washing and rinsing has also been highlighted in the cross-contamination of fruit and vegetables [26]. Cross-contamination can also occur at the cutting stage [27,28]. During slicing, either in processing facilities or in household kitchens, these bacteria can be transferred from the rind to fresh-cut pieces [29]. The internal environment of the cantaloupe, characterized by high sugar content and relatively low acidity (pH~6.7) [30], provides ideal conditions for the growth of *L. monocytogenes* and other foodborne pathogens [31]. If these contaminated fruits are not properly refrigerated, *L. monocytogenes* can proliferate to dangerous levels before any visible spoilage occurs [32]. Given the potential for contamination at any stage—from initial field production through to postharvest processing and final consumer handling—an expert committee of the Joint FAO/WHO Expert Meetings on Microbiological Risk Assessment (JEMRA) has underscored the need for a comprehensive production-to-consumption risk assessment to track and manage the introduction and propagation of *L. monocytogenes* in cantaloupes.

A recent review was conducted on existing quantitative risk assessment (QRA) models for *L. monocytogenes* in produce [33]. Most (9/13) QRA models examined focused on fresh or RTE leafy greens. Only one QRA model addressed cantaloupes [34]. This model considered the contamination of RTE cantaloupes in processing plants and aimed to assess the impact of improved hygiene and cold storage of the products. Yet, none of these QRA models incorporated critical contamination factors during primary production, including cropping systems, water, fertilizers, or irrigation methods and practices.

The JEMRA expert committee has thus recommended the development of a new QRA model that comprehensively encompasses the journey of cantaloupe from primary production to consumption, utilizing a modular approach supported by existing data. The JEMRA expert committee analyzed and provided recommendations on existing methods, data and models on the basis of the review carried out previously [33], and informed when gaps existed. Key stages to be incorporated would include growing, harvesting, cooling, washing, sanitizing, and dicing, alongside transportation, retail display, and consumer handling practices. The model must offer the flexibility to incorporate variables such as farming techniques, extreme weather conditions, climate change impacts, and varying market practices. It would consider potential cross-contamination at multiple points—ranging from preharvest environments (e.g., irrigation water and soil) to processing activities (e.g., equipment surfaces and pooling of fruits) and consumer-level interactions (e.g., contamination transfer during cutting).

Within this context, the main objective of this work was to construct a QRA model of longer scope for RTE cantaloupe than previously published, able to represent the survival, re- and cross-contamination, and potential growth of *L. monocytogenes* on/in cantaloupes from production to consumption. Widely based on published data, the model was structured to be able to assess the effectiveness of interventions and control measures, in line with the recent JEMRA recommendations [35,36]. The functionality of the QRA model proposed was illustrated by a reference scenario and by the development of what-if scenario analysis.

## 2. Materials and Methods

### 2.1. Exposure Assessment

**Figure 1:**
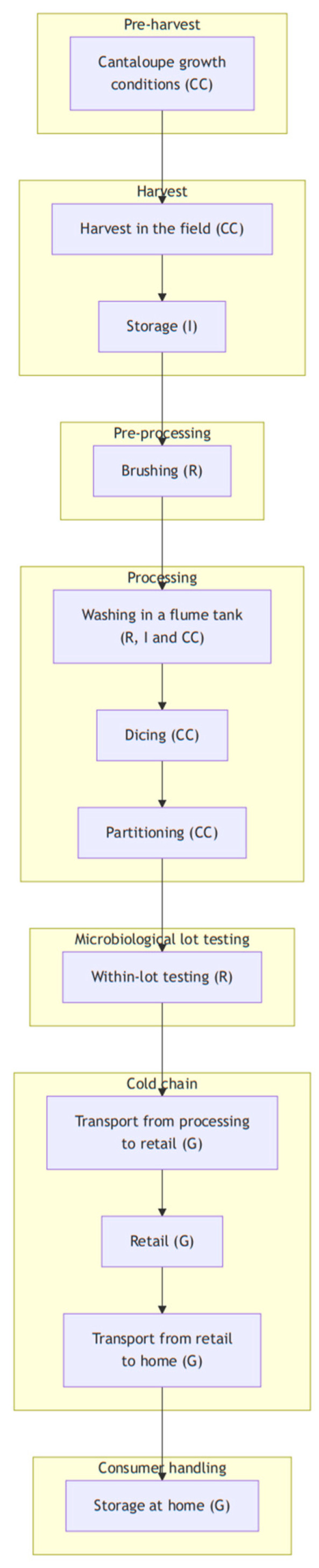
Flowchart for the RTE diced cantaloupe production process with indication of the modeled processes: CC: cross-contamination; I: inactivation; R: removal; G: growth.

The exposure assessment model for RTE cantaloupes comprises seven modules (Figure 1). The “Preharvest” module covers practices related to crop growth before harvest. It incorporates the use of barriers (mulch, foil) against soil contaminants by minimizing direct contact between crops, and the soil growing of cantaloupe was with or without soil barriers (i.e., mulch); also incorporated are the type of irrigation system (i.e., drip or overhead) and the use and type of soil amendments (e.g., non-composted organic fertilizers). These choices can impact the microbial environment around the crops and influence the potential risk of contamination. The “Harvest” module focuses on the hygiene management of food contact surfaces to minimize contamination during harvest. The “Pre-Processing” module addresses the management of brushing and washing, which is essential for removing contaminants from the surface of the cantaloupes. However, poor maintenance of the microbiological quality of the washing water can become a source of contamination. The “Processing module” emphasizes the importance of food contact surface hygiene and the potential transfer of contamination from the rind to flesh. The “Cold Chain Storage” module refers to the control of storage time and temperature during transport to retail to inhibit the growth of *L. monocytogenes*. Finally, the “Consumer Handling” module covers how the RTE cantaloupe is handled by consumers, with an emphasis mainly on transport from retail to consumers’ homes and storage at home (i.e., time and temperature). Each stage was modeled as a stochastic function estimating microbial prevalence and quantity based on fundamental processes [37] such as microbial growth, death, partitioning, removal or cross-contamination, according to the stage’s purpose. Additionally, a seventh module, “Microbiological Lot Testing,” is included to assess the effectiveness of microbial testing strategies in identifying and rejecting contaminated batches before distribution, thereby reducing the overall risk.

Table 1 summarizes the modules, the sequence of stages and processes they consisted of, the assumption and data sources employed, and the corresponding functions programmed in the R software (version 4.4.1).

#### 2.1.1. Preharvest Module

**Data and assumptions:**

The preharvest module for cantaloupe addresses agricultural practices and the potential contamination of *L. monocytogenes* from environmental sources, such as soil and irrigation water.

Factors such as growing cantaloupes using mulch as a soil barrier and the likelihood of soil contamination due to its ground-level growth are incorporated into the model. The prevalence of *L. monocytogenes* in agricultural soils varies widely (0.0 to 19.0%; see Table 2) and is influenced by factors such as the use of animal manure, soil conditions (e.g., pH, temperature), and irrigation methods [10,18,38,39,40,41,42,43,44,45]. Table 3 lists the effect of various risk factors on the soil prevalence of *L. monocytogenes*, as identified in the scientific literature. The survival of *L. monocytogenes* in soil is not significantly affected by manure type but varies depending on soil contamination methods [43,45]. Water sources such as municipal, rain, ground, and especially surface water also present variable contamination risks [46,47]. Additionally, fields located near pastures or water bodies face higher contamination risks, with surrounding land use, topography, and climate further affecting the risk [36,48]. Lastly, the model considers the natural decline in *L. monocytogenes* on cantaloupe rind before harvest [20,49].

Table 4 presents data on the occurrence of *L. monocytogenes* in irrigation water used for crop production. Three studies have quantified *L. monocytogenes* concentrations in positive water samples. Sharma et al. [50] and Acheamfour et al. [51] reported very low levels of *L. monocytogenes* in surface water sources, including creeks, ponds, tidal brackish river, and non-tidal fresh rivers, with MPN values ranging from <0.03 to 11 MPN/L. In contrast, Iwu et al. [52] reported higher mean concentrations of *L. monocytogenes* in irrigation water samples from 19 agricultural sites in South Africa (mean: 1196 CFU/100 mL; range: 0–5667 CFU/100 mL).

**Table 1:** Sequence of stages, microbial processes represented, data sources, assumptions and corresponding functions coded in R for the construction of the exposure assessment model of *Listeria monocytogenes* (LM) in RTE cantaloupe.

Module	Stage	Microbial Process	Assumptions	Sources	Function in R
Preharvest	Soil and irrigation water contamination	Contamination	The function assumes cantaloupe contamination through soil and irrigation water, with soil and water contamination characteristics such as the prevalence (pSoil and pIrrig) and distributions of *L. monocytogenes* concentration in soil and water as inputs. Risk factors such as irrigation prior to harvest, the use of organic fertilizer, or the use of a soil barrier (mulch) are included. It makes use of the outputs of caIrrig2rind and caSoil2rind sub-functions (see below).	[38,53,54,55]	caPrimaryProduction
	Irrigation water to rind contamination	Contamination	The function evaluates the contamination of cantaloupes through irrigation water only. It considers water contamination characteristics: prevalence (pIrrig) and concentration (cIrrig), which have to be chosen by the user based on existing data. cIrrig is conditional to water sources contaminated with *L. monocytogenes*.	[38,53,54,55]	caIrrig2rind
	Soil-to-rind contamination	Contamination	The function evaluates the contamination of cantaloupes through soil only. It considers soil contamination characteristics such as prevalence (pSoil) and concentration (cSoil) and the quantity of soil deposited on the cantaloupe rind. pSoil is conditional to risk factors such as irrigation before harvesting and the use of organic fertilizer, affecting pSoil with associated odds ratios (F_irrig_rain and fManure). It also takes into account the proportion of fields grown with a barrier (pFoil) and the reduction fraction of the quantity of soil transferred to the rind when a barrier is used (rFoil).	[18,56]	caSoil2rind
Harvest	Contamination during harvest	Cross-contamination	The function simulates cross-contamination that might occur at the moment of harvesting from elements such as conveyors, crates, or plastic surfaces. It can be used for cantaloupes harvested in farms (intended for RTE and sold as whole cantaloupe in formal retail). Parameters such as the probability of cross-contamination (probCCH) and the variability in the transfer coefficient (trMean, trSd) help to assess the transfer.		caHarvestCC
	Holding time post-harvest	Survival	The function simulates the survival of *L. monocytogenes* on cantaloupe rind during post-harvest holding time or during any short storage before cantaloupes are washed in the packinghouse. It calculates the decline in *L. monocytogenes,* assuming no growth on surfaces because any injury to the rind that would promote growth would be recent and the holding time too short for significant growth. The function allows defining a probability that the lot is kept at cold temperatures (4–10 °C).		caHoldingTime
Pre-processing	Brushing cantaloupes	Removal	The function caBrush() models the removal of bacteria during the brushing or scrubbing step of cleaning cantaloupes. It requires the mean log_10_ reduction due to brushing as an input, which quantifies how effectively bacteria are removed from the rind during this cleaning step.		caBrush
Processing	Flume tank cross-contamination	Cross-contamination	The caFlumeTankCC function simulates the potential contamination of cantaloupe when in direct contact with contaminated water in a flume tank. It accounts for four possible scenarios: cross-contamination in lots already contaminated, re-contamination in lots not previously contaminated, and scenarios where no cross-contamination occurs, regardless of initial lot status.		caFlumeTankCC
	Dicing of cantaloupe	Cross-contamination	The caDicing function simulates the transfer of *L. monocytogenes* from rind to flesh during the dicing of cantaloupe in a processing environment. It is assumed that each cantaloupe is separately diced, and if contaminated on the rind, a fraction of *L. monocytogenes* cells is transferred to the diced pieces. The function does not consider cross-contamination from dicing machines or knives.	[29,57]	caDicing
	Partitioning	Cross-contamination	The caPartitioningCC() function simulates the potential cross-contamination of cantaloupes during dicing and partitioning into packed units. It accounts for four possible scenarios of contamination involving sublots already contaminated or not. The algorithm also models the random distribution of *L. monocytogenes* from a contaminated sublot of diced cantaloupe into packed units using a dispersion factor, indicating the heterogeneity in the distribution of cells among pack units.	[58,59]	caPartitioningCC
Microbiological lot testing	Microbiological testing of RTE cantaloupe	Removal	The caTesting() function simulates the microbiological testing of RTE cantaloupe samples from a lot or sublot. It models sampling and testing based on a defined sampling plan (two-class or three-class). The algorithm uses bootstrapping to estimate the probability of detecting contaminated lots and returns the output matrix either in the original lot or sublot arrangement, depending on the user’s choice.		caTesting
Cold Chain Storage	All stages after processing	Growth	Bacterial growth is estimated using the primary growth model of with a lag phase Baranyi and Roberts [60], taking into account temperature conditions and the initial physiological state of cells (q0).	A range of research and studies, including [31,61], and several others, provide the data and parameters used to calibrate the growth model under temperatures below 30 °C.	caGrowthBaranyi
	Transport from processing to retail	Growth	The caTrans2RetRTE() function simulates the growth of *L. monocytogenes* in RTE diced cantaloupe during cold transport to retail, utilizing the caGrowthBaranyi() function. It assumes uniform initial conditions for all RTE diced cantaloupe packs from each lot, including the same initial q0, transport temperature, and time.	[62,63]	caTrans2RetRTE
	Display of RTE diced cantaloupe packs at retail	Growth	The caRetRTE() function simulates the growth of *L. monocytogenes* in RTE diced cantaloupe during display at retail, using the caGrowthBaranyi() function. It assumes uniform retail conditions for all RTE diced cantaloupe packs, including the same lnQt (from the previous logistic stage), retail temperature, and sampled retail time. The Pert distributions represent the variability in retail time and temperature.	[64]	caRetRTE
	Transport of RTE diced cantaloupe packs from retail to home	Growth	The caRet2HomeRTE() function simulates the growth of *L. monocytogenes* in RTE diced cantaloupe during transport from retail to home, using the caGrowthBaranyi() function. The transportation time and temperature are sampled at the unit level to reflect the variability depending on the consumer. The algorithm uses a gamma distribution for the variability in transport time and a Pert distribution for transport temperature.	-	caRet2HomeRTE
Consumer handling	Storage of RTE diced cantaloupe packs at home	Growth	The caHomeRTE() function simulates the growth of *L. monocytogenes* in RTE diced cantaloupe during home storage. It samples home storage time and temperature at the unit level, depending on consumer practices. The input data includes lot-specific values of EGR5 and unit-specific values of lnQt from the previous stage. Pert distributions are used to represent the variability in home storage time and temperature.	[62,65,66]	caHomeRTE

**Table 2:** *L. monocytogenes* prevalence in soils of produce fields.

Country	Characteristics	Positive/Total (%Prevalence)	Source
Canada	Cultivated fields, 7 fields fertilized with animal manure in addition to inorganic fertilizer	1/13 (7.7)	[38]
Malaysia	Vegetable fields in traditional farming	4/21 (19.0)	[67]
USA	Organic/Irrigate/Manure/Compost Farm 1: no/no/yes/yes Farm 2: yes/yes/yes/yes Farm 3: no/yes/yes/no Farm 4: no/yes/no/no Farm 5: no/no/no/no (data broken down by farm not available)	16/178 (8.9)	[55]
France	Cultivated soils from France	9/53 (17.0)	[39]
Poland	Lands fertilized with manure Lands fertilized with artificial fertilizers Garden plots intensively fertilized with manure Wastelands	2/173 (1.2) 0/173 (0.0) 5/47 (10.6) 0/120 (0.0)	[40]
Austria	Soil types (humus, sand, and clay)	28/467 (6.0)	[68]
USA	Soil samples from spinach fields Low-risk fields High-risk fields	24/546 (4.4) 62/546 (11.4)	[18]

**Table 3:** Data on likelihood of *L. monocytogenes* being detected in soils of produce fields, expressed as an odds ratio (OR) by risk factor.

Source	Risk Factor	Description	OR	95% CI	*p*-Value
[55]	Manure	Last time manure was applied			
		Within 365 days	7.0	[3.1–15.4]	<0.001
		Over 365 days	0.6	[0.2–1.7]	0.381
		Not applied	1.0		
	Irrigation	Last time field was irrigated			
		Within 3 days	6.0	[2.0–18.1]	0.010
		4–7 days	1.2	[0.3–4.5]	0.793
		8–14 days	0.4	[0.1–2.0]	0.288
		Over 14 days/not irrigated	1.0		
	Soil cultivation	Last time soil was cultivated			
		Within 7 days	2.9	[1.1–8.6]	0.050
		8–14 days	1.4	[0.4–5.1]	0.660
		15–30 days	0.4	[0.1–1.7]	0.224
		Over 30 days	1.0		
[18]	Irrigation/rain	Time since irrigation/rain occurred			
		24 h	25	[5.7–99]	0.010
		48 h	2.5	[0.49–12]	0.27
		72 h	3.4	[0.74–15]	0.11
		144–192 h	1.0		
	Amount of irrigation water (mm) applied to field 2 days before sample collection ^1^		1.2	[1.1–1.3]	0.010
[48]	Areas within 37.5 m of surface water		3.0	[2.0–4.6]	<0.001
	Areas within 62.5 m of pasture		2.9	[1.4–6.0]	0.005

^1^ Effect of a 1-mm increase in irrigation water application on the odds of isolating *L. monocytogenes*.

**Table 4:** Data *on L. monocytogenes* in water environments ordered by prevalence.

Country	Type of Water	Positive/Total (% Prevalence)	Source
Austria	River and pond	0/68 (0.0)	[68]
USA	Engineered water	0/28 (0.0)	[56]
USA	Engineered water	0/14 (0.0)	[55]
Malaysia	Irrigation water of vegetable farms	0/15 (0.0)	[67]
India	River water	8/100 (8.0)	[69]
Switzerland	River, stream, inland canal	25/191 (13.1)	[53]
Canada	Rural and urban watersheds	56/329 (17.0)	[70]
USA	Pond, river used for irrigation	2/9 (22.2)	[56]
South Africa	Roof-harvested rain water	72/297 (22.0)	[71]
Canada	Surface (river)	32/134 (23.9)	[72]
USA	Surface water	48/146 (33.0)	[55]
USA	Lake, stream, river, pond	605/1405 (43.1)	[73]
South Africa	Irrigation canal and river	19/36 (52.8)	[74]
USA	Surface water for irrigation	33/52 (63.5)	[18]

**The R functions:**

The function caPrimaryProduction() assesses the contamination of cantaloupes through soil and irrigation water. This function calls two auxiliary functions, caSoil2rind() and caIrrig2rind(), specifically built for each source of contamination.

The auxiliary function caSoil2rind() evaluates the contamination of cantaloupes through soil. It considers initial soil contamination characteristics of prevalence *p*_Soil_ (based on Table 2, the default value of 0.089 was selected). The initial prevalence of *L. monocytogenes* in soil (*p*_Soil_) is replicated for each lot (*n*_Lots_). This replication serves as the base probability of contamination for each field without considering any specific agricultural practices.

The probability of contamination is then adjusted for the first risk factor: usage of organic manure. A binary array (*b*_manure_ in the R script) is created where each element is either 0 or 1, determined randomly based on the proportion of fields that receive organic manure (*p*_Manure_). For fields indicated to use organic manure, the base soil contamination probability (*p*_Soil_) is adjusted using an odds ratio (*f*_Manure_). This adjustment reflects the increased risk of contamination due to manure use. The adjusted prevalence (*p*_SoilManure_) accounts for the base prevalence (*p*_Soil_) increased by the odds ratio if manure is used. The odds ratio has been estimated at 7.0 (*f*_Manure_) when manure is applied within 1 year [55].

$$p_{SoilManure}=\frac{f_{Manure}\cdot p_{Soil}}{1-p_{Soil}+f_{Manure}\cdot p_{Soil}}$$

A second adjustment is performed for irrigation/rain events. Similarly, another binary array (*b*_irrig_raining_) is created to indicate which fields experience irrigation or rain up to two days before harvest, based on a given probability (*p*_IrrigRaining_). For those fields, the previously adjusted prevalence (*p*_SoilManure_) is further modified by another odds ratio (*f*_IrrigRaining_). This odds ratio has been estimated at 25 (*f*_IrrigRaining_) based on [18]. This step reflects the additional risk of contamination from irrigation or rain events. The final soil prevalence (*p*_SoilIrrigRaining_) thus incorporates both manure and irrigation/rain factors.

$$p_{SoilIrrigRaining}=\frac{f_{IrrigRaining}\cdot p_{SoilManure}}{1-p_{SoilManure}+f_{IrrigRaining}\cdot p_{Soil}}$$

The final step aggregates the adjusted prevalences across all fields to compute the simulated overall prevalence of soil contamination (*p*_SoilSim_). This value represents the average prevalence of contamination across all simulated lots, taking into account variations due to manure use and weather conditions.

Together with the global prevalence of fields with contaminated soil, the function caSoil2rind() returns a matrix (*N*_0_) for quantities of *L. monocytogenes* coming from contaminated soils per cantaloupe. The function first calculates the quantity of soil that adheres to each cantaloupe (*soil*_gain_). This is modeled as a triangular distribution to account for variability from one cantaloupe to another within each lot. The distribution parameters—minimum (*q*_SoilMin_ = 0.05 g), mode (*q*_SoilMode_ = 0.5 g), and maximum (*q*_SoilMax_ = 5 g) values—are established as initial values by experts to represent realistic variations, though they do not directly rely on existing empirical evidence.

$${soil}_{gain}\sim Triangular\left(q_{SoilMin},q_{SoilMode},q_{SoilMax}\right)$$

To model the effect of using soil barriers (such as foil, plastic mulch) to protect cantaloupes from soil contact, a binary decision for each lot (*soil*_Foil_) is generated based on the probability of using barrier (*p*_Foil_). For lots with barrier protection, the quantity of soil adhering to the cantaloupes is reduced by a factor (*r*_Foil_), which represents the effectiveness of the barrier in preventing soil contact.

$${soil}_{Foil}\sim Binom\left(n_{Lots},p_{Foil}\right)$$

$${soil}_{gain}={soil}_{gain}\cdot \left(1-{soil}_{Foil}\cdot r_{Foil}\right)$$

The concentration of *L. monocytogenes* in the soil (*c*_Soil_) is also modeled with a triangular distribution, reflecting variability from field to field. The parameters of this distribution are given by *c*_SoilLogMin_, *c*_SoilLogMode_, and *c*_SoilLogMax_, and represent the log_10_ CFU/g of soil using data from Dowe et al. [38], who reported a mean of 4.0 MPN *L. monocytogenes*/g soil, with a 95% confidence interval of <1.0–28 MPN/g.

$$c_{Soil}\sim Triangular\left(c_{SoilLogMin},c_{SoilLogMode},c_{SoilLogMax}\right)$$

Finally, for caSoil2rind() function, the amount of *L. monocytogenes* transferred to the cantaloupe rind (*N*_0s_) is calculated by multiplying the quantity of soil attached to the rind by the pathogen concentration in the soil. This product gives the raw number of pathogens per cantaloupe. To introduce the possibility of stochastic variation and account for the Poisson distribution of pathogens, a Poisson process is applied to simulate the actual number of *L. monocytogenes* cells on each cantaloupe (the number of cantaloupes per lot, *sizeLot*, can be defined by the users).

$$N_{0s}\sim Poisson\left({soil}_{gain}\cdot c_{soil}\right)$$

The auxiliary function caIrrig2rind() evaluates the contamination of cantaloupes through water.

The proportion of lots contaminated by irrigation water is given by the prevalence of contamination in the water used (*p*_Irrig_). Table 4 lists many estimates of prevalence in water environments. The estimate of 0.131 provided by Raschle et al. [53] has been chosen as default value for *p*_Irrig_. The function assumes that the amount of water deposited on the cantaloupe rind after the last irrigation (*water*_gain_) is expressed as a proportion of the cantaloupe weight (*canta*_weigtht_), and is sampled from a uniform distribution between a minimum value of zero (*p*_WaterGainMin_) and a maximum value of 0.004 (*p*_WaterGainMax_), taken from Richards and Beuchat [54].

$${water}_{gain}={canta}_{weight}\cdot Uniform\left(p_{WaterGainMin},p_{WaterGainMax}\right)$$

The distribution of the concentration of *L. monocytogenes* in irrigation water (*c*_Irrig_) is represented as a uniform distribution, using the minimum and maximum values from Sharma et al. [50], who reported <0.03 to 11 MPN *L. monocytogenes*/L of water.

$$c_{Irrig}\sim 10^{Uniform\left(c_{IrrigLogMin},c_{IrrigLogMax}\right)}$$

Finally, for caIrrig2rind() function, the amount of *L. monocytogenes* transferred to the cantaloupe rind (*N*_0i_) is calculated by multiplying the quantity of water remaining on the rind by the pathogen concentration in the irrigation water. A Poisson process is applied to simulate the actual number of *L. monocytogenes* cells on each cantaloupe.

$$N_{0i}\sim Poisson\left({water}_{gain}\cdot c_{Irrig}\right)$$

Then, the caPrimaryProduction() function computes the prevalence of contamination from the prevalence in the two sources (soil and irrigation water) considered as independent. The results are then combined to estimate the overall prevalence of contamination (*P*_0_) across the entire production process.

$$P_{0}=p_{fromsoil}+p_{fromirrig}-\left(p_{fromsoil}\cdot p_{fromirrig}\right)$$

The main function caPrimaryProduction() also returns the contamination matrix (*N*_0_) by computing and combining the pathogen loads from two contamination sources (soil and irrigation water) on cantaloupes, in a probabilistic and combinatorial manner.

It first calculates exclusive and joint probabilities of the three possible situations. The probability of observing only soil contamination (*p*_OnlySoil_) is calculated as the probability of contamination coming from soil while not coming from irrigation water. The probability of observing only water contamination (*p*_OnlyIrrig_) is the inverse scenario, where contamination comes exclusively from irrigation water. The third scenario corresponds to the probability of observing both soil and water contamination simultaneously (*p*_SoilandIrrig_).

$$p_{OnlySoil}=p_{FromSoil}\cdot \left(1-p_{FromIrrig}\right)$$

$$p_{OnlyIrrig}=p_{FromIrrig}\cdot \left(1-p_{FromSoil}\right)$$

$$p_{SoilIrrig}=p_{FromIrrig}\cdot p_{FromSoil}$$

Then the contamination matrix (N0) is populated with contamination data based on the above probabilities. When soil is the only source of contamination (*N*_OnlySoil_), a proportion (*p*_OnlySoil_) of the lots received the contamination outputs from caSoil2rind(). When irrigation water is the only source of contamination (*N*_OnlyIrrig_), a proportion of the lots (*p*_OnlyIrrig_) received the contamination outputs from caIrrig2rind(). For lots contaminated by the two sources the sum of soil and irrigation matrices were used to represent the combined contamination levels.

#### 2.1.2. Harvest of Cantaloupes

**Data and assumptions:**

Cantaloupes are hand-harvested and loaded onto transport vehicles via mobile conveyor belts, with loading either automated or assisted manually. Rough handling, such as throwing or dropping, can damage the rind, potentially allowing *L. monocytogenes*, present on surfaces, to penetrate and grow. After harvest, cantaloupes are typically transported to a packinghouse, where they undergo cleaning, washing and cooling. Given that transport times are usually under a few hours, the potential for growth of *L. monocytogenes* during this time was not considered relevant for this model.

During transport, there is also a risk of cross-contamination between cantaloupes and food contact surfaces. In packinghouses, fruits from different farms or fields may be mixed, but there was no data to support the inclusion of a transportation module in the model. Additionally, stem scars from harvesting and poor worker hygiene could contribute to contamination. However, based on expert opinions and the lack of quantitative data, these factors were not considered significant for the model.

Data were collected to prepare the two functions needed to describe the fate of *L. monocytogenes* on cantaloupes, the decline in *L. monocytogenes* on intact rind and the cross-contamination.

For the decline from harvest to processing, data from Ukuku and Fett [57] and Nyarko et al. [49] were used (Table 5). The bacteria are more prevalent on cantaloupes stored at 25 °C than at 4 °C, with studies confirming die-off on the rind surface under low temperatures.

**The R functions:**

The function caHarvestCC() simulates cross-contamination that might occur at the moment of harvesting from elements such as conveyors, crates or plastic surfaces.

It first considers the contamination of already contaminated lots (*P*_preharvest_). The function generates a matrix of probabilities (*p*cc_flag_) for each cantaloupe (*n*_Lots_ × *size*_Lot_), based on the binomial probability that cross-contamination occurs (*prob_CCH_*).

$${pcc}_{flag}\sim Binomial\left(n_{Lots}\cdot {size}_{Lot},{prob}_{CCH}\right)$$

The transfer from conveyors, crates or plastic surfaces depends on the transfer rate, which is assumed to vary from lot to lot (*TR*_i_). The parameters characterizing the variability in the transfer rate were taken from Hoelzer et al. [58].

$${TR}_{i}\sim Normal\left(-1.42,0.55\right),{TR}_{i}\leq 0$$

If a recontamination event occurs at the cantaloupe level (as determined by *pcc_flag_*), a fraction (*TR_i_*) of the bacterial load present on the contaminated equipment surface(s) (*N_plas_*) is transferred to the rind (*N_from-equip_*).

$$N_{from-equip}\sim Binomial\left({TR}_{i},N_{plas}\right)\cdot {pcc}_{flag}$$

The total number of *L. monocytogenes* (*N_post_*_harvest_) is then simply the sum of cells already present before harvest (*N*_prehavest_) and the ones transferred from equipment.

$$N_{postharvest}=N_{preharvest}+N_{from-equip}$$

The function also accounts for previously uncontaminated lots (probability 1 *− Ppreharvest*) that may become contaminated through contact with contaminated equipment. For each of these lots, the potential bacterial transfer (*N_from-equip_*) is evaluated. If *N*_from-equip_ is > 0, the load of the lots is simply equal to *N*_from-equip_ (as *N*_preharvest_ is null for this type of lot). The proportion of the batch with no cells transferred is thereafter noted *prob*_clean-batch_.

The overall prevalence of contamination in the batch is then reassessed:

$${p_{harvest}=p}_{preharvest}+\left(1-p_{preharvest}\right)\cdot \left(1-{prob}_{clean-batch}\right)$$

and, along with *N_postharvest_*, *p_harvest_* becomes a function’s output.

The caHoldingTime() function models the survival of *L. monocytogenes* on cantaloupe rind during post-harvest holding or short pre-wash storage in packinghouses. The algorithm focuses on the decline in *L. monocytogenes*, assuming that any rind injury from harvesting would prevent significant bacterial growth due to the brief holding period. Users can specify the probability *p*_Cooled_ that a batch of newly harvested cantaloupes is stored at cold temperatures (4–10 °C). If the batch is not stored at a cold temperature, the model assesses survival at room temperature (25 °C).

Survival data for *L. monocytogenes* on intact rinds of two cantaloupe cultivars at 4, 10, and 25 °C, sourced from Nyarko et al. (2016) [49] (see Table 5), were used. A non-linear mixed model using the two-parameter Weibull decay equation, with random effects by cantaloupe variety, revealed that decay rates at 25 °C differ significantly from those at 4 and 10 °C, which showed no substantial difference. This model segments temperatures into two categories: cool (4/10 °C) and ambient (25 °C), with a fixed *shape* parameter for each. Random effects allow for cultivar-specific variability in decay times (*D*), generating estimates for *Dmean*_410_, *Dsd*_410_, *Dmean*_25_, and *Dsd*_25_, which represent average decay and standard deviations for cool and ambient temperatures, respectively. To represent variability among cantaloupe cultivars, *D* values are sampled for each lot.

$$D_{T}\sim Norm\left(D_{meanT},D_{SDT}\right),D_{T}>0$$

psurvive=10−t24·DTshape

The number of *L. monocytogenes* on rinds of cantaloupes after the holding period (*t*) is then assessed according to the initial number present (*N*_harvest_) and the probability of survival of one bacterial cell (*p*_survive_),

$$N_{endharvest}\sim Binomial\left(N_{harvest},p_{survive}\right)$$

It is assumed that even after the holding period, no initially contaminated lot will be entirely free of *L. monocytogenes*, and thus, a minimum of one bacterial cell will be allocated to one cantaloupe from any contaminated batch. The prevalence of contamination in the batch is thus unchanged by the decline in *L. monocytogenes*. The proportion of contaminated batches remains unchanged, even if the concentration of *L. monocytogenes* decreases.

#### 2.1.3. Pre-Processing: Cleaning and Washing

**Data and assumptions:**

The cleaning and washing module addresses hygiene concerns associated with brushes used for dry cleaning and with washing equipment used for the spray or immersion washing of freshly harvested cantaloupe.

The first treatment in the processing facility was the cleaning of cantaloupes with a dry-brushing step. Abrasive brushing was found to reduce biofilms and to reduce the *L. monocytogenes* load by 1.4 log_10_ [75].

The second step involved washing cantaloupes through spray or immersion washing, as well as hydrocooling to remove field heat. Washing with water alone typically achieves reductions in L. monocytogenes on cantaloupe surfaces below 1.0 log_10_ CFU/cm^2^ [29,76,77]. The reduction in *L. monocytogenes* on the cantaloupe rind due to washing with water and sanitizers can be modeled using the data compiled in Table 6. Table 6 gathers literature data on the effectiveness of chemical sanitizers, including the mean reduction effect of washing and determinant features of the washing treatment, such as exposure time, temperature and sanitizer concentration. Nonetheless, in the function, only washing with water was considered.

The reductions reported in Table 6 are quite large, most probably due to the use of optimal lab conditions. In industrial settings, maintaining proper application conditions is challenging, significantly reducing the effectiveness of sanitizers in preventing cross-contamination [26,78]. In the best situation, the washing would not contribute to an increase in contamination; in the worst situation, flume tank washing could increase contamination through contaminated water.

**The R functions:**

Three functions were prepared to account for the brushing effect and the two alternate washing steps (ideal situation with potential population decrease, and one with potential cross-contamination).

The function caBrush() models the removal of bacteria during the brushing or scrubbing step. This function takes into account the effectiveness of the cleaning step, quantified as a mean log_10_ reduction in bacteria, that users need to provide as an input parameter. The function processes a list containing an initial matrix of bacterial counts on the cantaloupe rinds and the prevalence of contamination in harvested lots. This reduction can vary at lot level or at cantaloupe level, depending on how *logDecBrush* is defined (either as a single value, a vector per lot, or a matrix per cantaloupe).

$$N_{afterbrush}\sim Binomial\left(N_{endharvest},10^{-LogDecBrush}\right)$$

The function caFlumeTank() simulates three events taking place during flume tank washing: the washing of bacteria off the cantaloupe rind at a washing efficiency of *logDecWash*, the elimination of bacteria in the water containing sanitizers at a sanitizing efficiency of *log10SaniWash*, and the redistribution of survivors on the cantaloupe rind (characterized by the *b* parameter).

For the first event (washing of the bacteria from the rind to water), the number of bacteria remaining on the rind is calculated according to the parameter *logDecWash*, the reduction in log_10_ attained by washing.

$$N_{NotWash}\sim Binomial\left(N_{afterbrush},10^{-LogDecWash}\right)$$

The quantity of *L. monocytogenes* in the water (*N*_wash_) is given by

$$N_{wash}=N_{afterbrush}-N_{NotWash}$$

Some of the *L. monocytogenes* in water will be inactivated by the presence of a sanitizer. The log_10_ reduction associated with the sanitizer (*logDecSani*) helps determine the number of *L. monocytogenes* that will be redistributed onto the cantaloupe rinds (*N*_toDistribute_)

$$N_{toDistribute}\sim Binomial\left(N_{wash},10^{-LogDecSani}\right)$$

Finally, the redistribution of survivors on the cantaloupe rind was modeled according to a Dirichlet–multinomial process. The parameter to assess the dispersion factor representing the clustering of surviving cells during redistribution is *b*. A value of *b* higher than 1 should be used to assume random homogeneous distribution. The redistribution is handled computationally by first drawing proportions (***p***) from a Dirichlet distribution parameterized by *b*, which gives the fraction of surviving bacteria that each cantaloupe receives. Then, a multinomial distribution is used to allocate the actual counts of bacteria to each cantaloupe (*N*_out_) based on these proportions.

$$p\sim Dirichlet\left(b\right)$$

where ***b*** is a vector of *b*/*size_Lot_* repeated *size_Lot_* time and $N_{out}\sim Multinomial\left(N_{toDistribute},p\right)$.

Finally, the total number of *L. monocytogenes* per cantaloupe rind (*N*_endwashing_) is the sum of the cells that remained on the rind plus the redistributed ones.

$$N_{endwashing}=N_{NotWash}+N_{out}$$

A second function was prepared for modeling the effect of washing cantaloupes. The caFlumeTankCC() function simulates potential contamination scenarios for cantaloupes when they come into direct contact with contaminated water in a flume tank. The cross-contamination algorithm considers four different scenarios [79]: cross-contamination in lots that are already contaminated, contamination in lots that were initially not contaminated, the absence of cross-contamination in lots that were already contaminated, and no cross-contamination occurring in lots that were not initially contaminated. The probabilities of each scenario are calculated according to the initial prevalence (*P*_harvest_) and the probability that the flume tank water is contaminated (*prob_CCW_*):

$$P\left({CantaLot}_{Pos}\_{CC}_{Negi}\right)=P_{harvest}\times \left(1-{Prob}_{CCW}\right)$$

$$P\left({CantaLot}_{Pos}\_{CC}_{Posi}\right)=P_{harvest}\times {Prob}_{CCW}$$

$$P\left({CantaLot}_{Neg}\_{CC}_{Posi}\right)=\left(1-P_{harvest}\right)\times {Prob}_{CCW}$$

The status of every lot is then randomly sampled from the probabilities [$P\left({CantaLot}_{Pos}\_{CC}_{Negi}\right),P\left({CantaLot}_{Pos}\_{CC}_{Posi}\right),P\left({CantaLot}_{Neg}\_{CC}_{Posi}\right)]$. This strategy is adopted to maintain the dimension [r, c] of the input contamination matrix $N_{harvest}$. The number of *L. monocytogenes* transferred (N_added_) depends on the concentration in the flume tank water (C_water_) and the value of the fraction of water gain (mL) (p_WaterGain_) relative to the cantaloupe weight in g,

$$C_{water}\sim Pert\left(logWaterMin,logWaterMode,logWaterMax\right)$$

$$N_{added}=C_{water}\cdot p_{WaterGain}\cdot {canta}_{Weight}$$

Then, the values of *N*_added_ and the total contamination on rinds before washing (*N_tot i_*,) are summed in order to determine the numbers of *L. monocytogenes* in the lot *i* after the potential recontamination event (*N_tot postWaterCC i_*), according to the following:

$$N_{tot\;postWaterCC\;i}=\left\{\begin{array}{cc} N_{tot\;i} & i\in {CantaLot}_{Pos}\_{CC}_{Neg}\\ N_{tot\;i}+N_{added\;i} & i\in {CantaLot}_{Pos}\_{CC}_{Pos}\\ N_{added\;i} & i\in {CantaLot}_{Neg}\_{CC}_{Pos} \end{array}\right.$$

Next, the partitioning algorithm randomly distributes the total numbers of cells *N_tot postWaterCC i_* from a contaminated lot onto all cantaloupe rinds present in the lot, following a Dirichlet–multinomial process according to a dispersion factor (*b*_w_) representing the clustering of cells during flume tank washing:

$$p_{w}\sim Dirichlet\left(b_{w},n_{Lots}\right)$$

where ***b_w_*** is a vector of *b_w_*/*size_Lot_* repeated *size_Lot_* times.

$${N_{washedrind}}_{i}\sim Multinomial\left(N_{totpostCC},p_{w}\right)$$

#### 2.1.4. Processing

**Data and assumptions:**

This module addresses the contamination of the RTE diced cantaloupe, considering two main sources. The first represents contamination from the cantaloupe rind to the flesh during slicing and dicing. Previous studies mimicking an industrial processing line and home preparation procedures for fresh-cut cantaloupe have shown that *L. monocytogenes* readily transfer from the rind to the edible cantaloupe flesh during slicing and cutting, with a positive correlation between the contamination level on the rind and the numbers of cells transferred [29,80,81]. Patil [81] conducted an experiment to measure the penetration of *L. monocytogenes* into cantaloupe flesh from contaminated rinds. Cantaloupes with an initial rind contamination of 5 log_10_ CFU/cm^2^ were stored at 4 °C and 30 °C for 24 h, then sampled using a cork borer to create 25 mm long cylinders. The bacteria’s penetration was measured at various depths up to 25 mm. The results showed that the transfer of bacteria from the rind to the first 5 mm of flesh ranged between 2.8 and 3.6 log_10_ CFU/cm^2^ at 4 °C and 1.9 and 4.0 log_10_ CFU/cm^2^ at 30 °C. No significant differences were observed in the bacterial counts transferred at different depths or between the two storage temperatures, indicating that all depths of the edible flesh were equally susceptible to contamination regardless of the storage condition.

Other studies aimed to quantify the transfer of *L. monocytogenes* from cantaloupe rind to fresh-cut pieces [20,29]. Inoculated whole cantaloupes stored at 4 °C were cut into four sections, with each section further diced into 3 cm cubes before rind removal. The overall transfer rate data are summarized in Table 7. As few as 150 bacterial cells per cm^2^ on the cantaloupe rind were sufficient to contaminate the edible flesh during cutting [81]. Transfer rates were generally between 0.08 and 1.18%.

The second relevant source of contamination is the cross-contamination of cantaloupes with *L. monocytogenes* from slicing and dicing equipment as well as other food contact surfaces [81]. The modeling approach used for this process was similar to the one used for cross-contamination during harvesting. The transfer rate was taken from [58]. Based on environmental data established for *Listeria* spp. [27], the initial concentration on food contact surfaces was estimated at 0.25 CFU per site (25 cm^2^). Given this surface area and considering the estimated contact area of a cantaloupe with the contaminated surface (5 cm^2^), the expected *L. monocytogenes* load transferred to the cantaloupe is approximately 9 CFU.

**The R function:**

The function caDicing() models the transmission of *L. monocytogenes* from the rind to the flesh when cantaloupes are diced in a processing environment. It operates under the assumption that each cantaloupe is diced individually, and if the rind is contaminated, a certain percentage of *L. monocytogenes* cells are transferred to the diced pieces. It is important to note that this function does not account for any potential cross-contamination that might occur from the dicing equipment or knives. The function returns the matrix *N*_dicing_ organized according to sublot configurations, where each row represents a sublot and each column corresponds to the number of cantaloupes processed within that sublot. This structure is used because the size of production lots may differ from the size of processing lots. Thus, the numbers and prevalence of contaminants are reported on a sublot basis (processing lot size). The variability in the transfer rate of *L. monocytogenes* from the rind to the flesh (CFU/g of diced cantaloupe per CFU/cm^2^ of rind) is modeled using a Pert distribution. This modeling utilizes data digitized from studies by Ukuku and Fett [57] and Ukuku et al. [29]. The transfer rate (*TR*_rind2flesh_) is expressed as a percentage (CFU/g of diced pieces per CFU/cm^2^ of rind) × 100 and is described using by the parameters *min*_TR_, *mode*_TR_, and *max*_TR_.

$${TR}_{rind2flesh}\sim PERT\left({min}_{TR},{mode}_{TR},{max}_{TR}\right)$$

For calculating the number of *L. monocytogenes* bacteria transferred from the rind to the flesh of cantaloupes during the dicing process (*N*_trans_), the density of bacteria per square centimeter on the rind of each cantaloupe within the sublots is assessed. This is achieved by dividing the total bacterial count on the rind (*N*_sublots_) by the surface area of the cantaloupe (*cantaSurface*). Following this, the transfer rate (*TR*_rind2flesh_) is applied to this density. Defined as a percentage, this rate quantifies the effectiveness of the bacterial transfer from the rind to the flesh during dicing. For calculation purposes, it is adjusted by dividing by 100. After applying the transfer rate, the resulting value, which indicates the number of bacteria transferred per gram of diced cantaloupe, is then scaled up by the total weight of the diced pieces produced from one rind-free cantaloupe (*cantaRindFree*).

$$N_{trans}=cantaRindFree\cdot {TR}_{rind2flesh}\cdot \frac{N_{sublots}}{cantaSurface}\cdot \frac{1}{100}$$

The overall prevalence is adjusted taking into account that, in some lots, no *L. monocytogenes* are transferred (*P_i0_*):

$$P_{diced}=P_{beforedicing}\cdot \left(1-P_{i0}\right)$$

The function caPartitioningCC()models the possible cross-contamination of cantaloupes in scenarios where they come into direct contact with dicing machines or knives. This function also handles the distribution of diced cantaloupes from a processing lot (sublot) into packed units. The cross-contamination algorithm evaluates four scenarios: contamination and no contamination occurring in both already contaminated and initially uncontaminated sublots, with probabilities calculated for each event. Following contamination assessment, the partitioning algorithm allocates the total number of bacterial cells from a contaminated sublot into individual packed units. This distribution considers the dispersion factor *b_i_*, a parameter of the beta distribution, which measures the extent of cell clustering within the bulk of diced cantaloupes in the sublot and reflects the variability in cell numbers among the packed units.

If the recontamination event takes place, at a probability *Prob*_CCDice_, a fraction (*TR_Dicer_*) of the numbers of cells on the contaminated equipment surface(s) (*N_Dicer_*) is transferred to the diced pieces of cantaloupe (*N_from-dicer_*). The algorithm considers that, if the lot of RTE diced cantaloupe is not contaminated with *L. monocytogenes* (actually not represented in the contamination matrix), it may become contaminated from equipment; if the lot is contaminated, its load will increase or remain the same depending on the recontamination event happening or not, respectively. This raises three possible scenarios of contaminated lots: (1) no recontamination occurring in lots already contaminated (*Dice_Pos__CC_Neg_*); (2) recontamination occurring in lots already contaminated (*Dice_Pos__CC_Pos_*); and (3) recontamination occurring in lots that were not contaminated (*Dice_Neg__CC_Pos_*). The probability of each scenario taking place for every lot *i* is calculated as

$$P\left({Dice}_{Pos}\_{CC}_{Neg\;i}\right)=P_{diced}\cdot \left(1-{Prob}_{CCDice}\right)$$

$$P\left({Dice}_{Pos}\_{CC}_{Pos\;i}\right)=P_{diced}\times {Prob}_{CCDice}$$

$$P\left({Dice}_{Neg}\_{CC}_{Pos\;i}\right)=\left(1-P_{diced}\right)\times {Prob}_{CCDice}$$

The status of every lot is then randomly sampled from the probabilities [$P\left({Dice}_{Pos}\_{CC}_{Neg\;i}\right),\;P\left({Dice}_{Pos}\_{CC}_{Pos\;i}\right),\;P\left({BDice}_{Neg}\_{CC}_{Pos\;i}\right)$]. The extent of recontamination in number of cells per lot *i* is computed as

$${TR}_{Dicer\;i}\;\sim \;Normal\;\left(-1.42,\;0.52\right),\;\;\;\;{TR}_{i}\leq 0$$

$$N_{from-dicer\;i}\;\sim \;Binomial\left(N_{Dicer},\;10^{{TR}_{Dicer\;i}}\right),\;\;\;\;N_{Dicer\;i}>0$$

where *N_Dicer_* can be understood as the total load of *L. monocytogenes* cells on the surface of the blades of the dicing machine and/or packaging machine in contact with the diced pieces of cantaloupe. Next, the value of *N_from-Dicer i_* is added to *N_tot i_*, in order to determine the numbers of *L. monocytogenes* in the lot *i* after the potential recontamination event (*N_tot postCC i_*), according to the following:

$$N_{tot\;postCC\;i}=\left\{\begin{array}{cc} N_{tot\;i} & i\in {Dice}_{Pos}\_{CC}_{Neg}\\ N_{tot\;i}+N_{from-equip\;i} & i\;\in \;{Dice}_{Pos}\_{CC}_{Pos}\\ N_{from-equip\;i} & i\in {Dice}_{Neg}\_{CC}_{Pos} \end{array}\right.$$

Next, the partitioning algorithm randomly distributes the total number of cells *N_tot postCC i_* from a contaminated lot into *c* packed units, following a Dirichlet–multinomial process:

$$p\;\sim \;Dirichlet\left(b_{i},n_{Lots}\right)$$

where ***b_i_*** is a vector of *b_i_*/*size_Lot_* repeated *size_Lot_* times.

$${N_{pack\_c}}_{\;i}\;\sim \;Multinomial\left(N_{tot\;postCC\;i},p\right)$$

The resulting matrix *N_pack_c_* contains the numbers of *L. monocytogenes* cells in the packs of diced cantaloupe in contaminated lots. Finally, the mean prevalence of contaminated lots after packaging *P_Pack_* is updated.

$$P_{Pack}=1-\left(1-P_{diced}\right)\times \left(1-{Prob}_{CCDice}\right)$$

#### 2.1.5. Microbiological Lot Testing

**Data and assumptions:**

The QRA model enables the microbiological testing of food unit samples taken from a lot, according to a two-class or a three-class mixed sampling plan [82]. In the commonly employed two-class plan for *L. monocytogenes*, a predetermined number *n* of units is randomly selected from each lot for testing. Each selected unit undergoes an enrichment assay using *g* grams of the sample, and the lot is considered unacceptable if more than *c* units test positive. In contrast, the three-class mixed sampling plan not only tests but also enumerates the sampled units. Here, a lot is rejected if more than *c* units are positive or if any unit has a microbial concentration exceeding a set threshold *M*. Enumeration tests are typically performed only on samples that test positive in the initial detection, involving the direct plating of a specific amount *g_TestedEnum_* from the same sample.

**The R function:**

The function caTesting() receives the outputs of the function caPartitioningCC(): *n*, the number of tested units; *g*, the sub-sample weight in grams used for detection; *c*, the number of positive samples accepted (two-class or three-class mixed plan); *M*, the maximum limit concentration; *p_lot tested_*, the proportion of tested lots; *Se*, the probability of the test (enumeration or detection) to detect, independently, each bacteria present in a sample; and *g_TestedEnum_*, the sub-sample weight in grams used for the enumeration assay.

Assuming perfect homogenization in the sample, each of the bacteria present in each of the *r* × *c* units of *Unit_size_* weight has a probability of being present in the *g* grams of the sub-sample and detected with probability equal to $Se\times {g/Unit}_{size}$. The number of bacteria detected in the detection assay is then as follows:

$$N_{detected\;ij}\;\sim \;Binomial\left(N_{Pack\;ij}\;,Se\times {g/Unit}_{size}\right)$$

and the detection test is positive if $N_{detected\;ij}>0$.

If the sampling plan is a 3-class plan, an enumeration test (direct plating) is performed. The number of bacteria enumerated in the sample is as follows:

$$N_{enumerated\;\;ij}\;\sim \;Binomial\left(N_{Pack\;ij}\;,Se\times {g_{TestedEnum}/Unit}_{size}\;\right)$$

and the estimated concentration is $N_{enumerated\;ij}/g_{TestedEnum}$ CFU/g. The algorithm assumes that the enumeration is performed only on samples detected as positive.

In order to evaluate the probability of each of the lots to be rejected, 1000 (by default) Monte Carlo samples of *n* samples are generated for each lot and, for each of these Monte Carlo samples, the microbiological criteria are applied (i.e., in a 2-class plan, the test is positive if >*c* samples among *n* are detected, whereas in a 3-class plan, the test is positive if >*c* samples among *n* are detected or if at least one sample has an estimated concentration >*M* CFU/g). The mean number of times the lot is rejected among the Monte Carlo samples multiplied by the probability for the lot to be tested is an estimate of *P_pos i_*, the probability of lot *i* to be rejected.

Contaminated lots detected after testing are not removed from the matrix, and therefore, the input matrix *N_pack_* is returned unchanged. The function caTesting() only updates the prevalence outputs.

#### 2.1.6. Cold Chain During Transport to Retail

RTE diced cantaloupe is typically refrigerated at retail. However, time–temperature measurements reveal that cold storage in retail display cabinets is often insufficient, frequently exceeding recommended limits [83]. Derens-Bertheau et al. [64] observed that about 31% of temperature measurements for various food products in France exceeded 6 °C, with retail display temperatures ranging from 1.4 °C to 9.8 °C, averaging 5.6 °C. Similarly, another study [84] recorded average temperatures of 4.1 °C and 3.5 °C for fresh-cut lettuce in winter and summer, respectively.

Herein, the impact of temperature on *L. monocytogenes* growth in cantaloupe was assessed using predictive microbiology models. The aim was to develop secondary models for lag phase duration and growth rate based on the published data presented below. Scolforo et al. [85] determined lag phase duration of *L. monocytogenes* serovar 1/2b inoculated in the pulp of Canary melons stored at different temperatures. A Q0 parameter characterizing the physiological state of the cells in cantaloupe was derived from these experiments.

For the growth rate, we used the challenge study of *L. monocytogenes* 4b, 1/2a, and 1/2b carried out by Fang et al. [61], who assessed the effect of serotypes on growth rates across all temperature conditions. They did not find any serotype effect or interaction with temperature, and subsequently, they determined the cardinal parameters for temperature of *L. monocytogenes* in fresh-cut cantaloupe (T_min_ = 1.90 °C, T_opt_ = 38.3 °C, T_max_ = 45.7 °C, μ_opt_ = 0.975 h^−1^). Table 8 compiles the growth rate estimates for cantaloupe from various studies, including the results from squash (a fruit also from the *Cucurbitaceae* family; Farber et al. [86]), since its estimates are comparable to those of cantaloupe.

The differential form of the Baranyi and Roberts model [60] was employed to predict the growth of *L. monocytogenes* in cantaloupe, providing a value for Q_0_ to assume the presence of lag phase.

$$\frac{dN}{dt}=\mu_{max}\frac{Q}{1+Q}\left(1-\frac{N}{10^{MPD}}\right)N$$

$$\frac{dQ}{dt}=\mu_{max}Q$$

**Figure 2:**
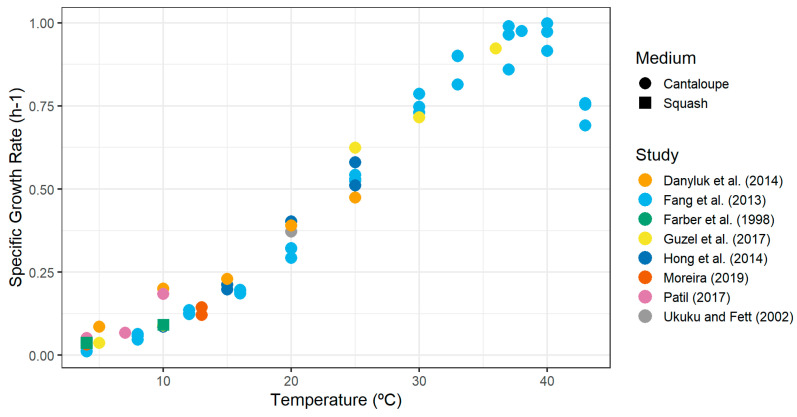
Specific growth rate estimates of *L. monocytogenes* in cantaloupe flesh against temperature, extracted from the literature. Refer to Table 8 for data sources [31,57,61,76,81,86,87,88].

Figure 2 plots the specific growth rate estimates against storage temperature. The values obtained for temperatures below 30 °C were used to prepare a secondary model for temperature.

For temperature, data were collected for the three stages of the logistic chain: transport from processing to retail, storage at retail, and transport from retail to the consumer home.

Fresh-cut cantaloupe is usually transported in refrigerated trucks, whose temperature should be lower than 7 °C [89]. A Pert distribution is used to account for the temperature variability. Considering the values encountered for the storage of produce, the temperature is assumed to be between 3 °C and 10.3 °C with a mean of 5 °C [62,63].

From retail to home, the temperature typically rises, as the consumer usually does not take measures to prevent an increase in surface temperature. Risk assessments for *L. monocytogenes* usually account for this stage. For example, Pouillot et al. [90] considered a mean temperature of cold-smoked salmon of 10.4 °C during transport. Additionally, several risk assessments have reported an average transport time from retail to home of 43 min (with a 19 min standard deviation) [63,66].

As the shelf life of RTE diced cantaloupe is quite short, at usually less than 5 days [91], the packs are expected to stay a day on retail shelves.

**The R functions:**

Three functions were developed for modeling the growth of *L. monocytogenes* from processing to the consumer home. They all rely on the auxiliary function caGrowthBaranyi(). As inputs, this function takes time and temperature for a logistic stage, the levels of initial contamination at the beginning of this stage, and its physiological state ($Q_{0}$). It returns the level of contamination at the end of the stage and its new physiological state (updated $Q_{0}$).

From user inputs, this function first provides an estimation of the growth rate according to temperature

$$\mu_{max}={EGR}_{5}\cdot {\left(\frac{\left(T-T_{min}\right)}{5-T_{min}}\right)}^{2}$$

where EGR_5_ is the growth rate (in h^−1^) of *L. monocytogenes* in cantaloupe flesh at 5 °C and $T_{min}$ is the nominal minimum temperature for the growth of *L. monocytogenes* in cantaloupe flesh.

Then the function uses *µ*_max_ and the time and duration of the logistic stage to assess the final level. The maximum population in the RTE cantaloupe pack depends on the unitSize and the maximum population density (MPD) of *L. monocytogenes* (set by default to 8 log_10_ cfu/g). The primary model of Baranyi and Roberts [60] in static environmental conditions is given by

$$A\left(t\right)=t+\frac{1}{\mu_{max}}\cdot ln\left(\frac{exp\left(-\mu_{max}\cdot t\right)+Q_{0}}{1+Q_{0}}\right)$$

$$ln\left(N\left(t\right)\right)=ln\left(N\left(t=0\right)\right)+\mu_{max}\cdot A-ln\left(1+\frac{exp\left(-\mu_{max}\cdot A\right)-1}{exp\left(ln\left(10^{MPD\cdot unitSize}\right)-ln\left(N\left(t=0\right)\right)\right)}\right)$$

The parameters used for the three functions thereafter were fitted according to the data presented in Figure 2 (below 30 °C): T_min_ = −2.02 °C (standard error = 0.576 °C) and EGR_5_ = 0.036 h^−1^ (standard error = 0.004 h^−1^).

Finally the caGrowthBaranyi() returns the physiological state value at the end of the logistic state:

$$ln\left(Q_{t}\right)=ln\left(Q_{0}+\mu_{max}\cdot t\right)$$

The first function of the logistic chain is the caTrans2RetRTE() function. It models the growth of *L. monocytogenes* in RTE diced cantaloupe during cold transportation to retail outlets, utilizing the auxiliary caGrowthBaranyi() function for growth dynamics. This function assumes that all cantaloupe packs in a lot experience the same initial physiological state of cells (Q_0_), consistent transportation temperature (Temp), and transportation duration. The variability in the physiological state parameter, ln(Q0), was represented by a normal distribution with a mean (lnQ0Mean) and standard deviation (lnQ0Sd) of −0.097 and 0.0640, respectively, according to [85].

Similarly, the variability in the specific growth rate at 5 °C (EGR_5_) is modeled using a normal distribution with parameters meanEGR_5_ = 0.03557288 h^−1^ and sdEGR_5_ = 0.004 h^−1^.

Additionally, the function uses Pert distributions to account for lot-specific differences in transport time (time_2Ret_) and temperature (temp_2Ret_) for this logistic step.

$${time}_{trans2Ret}\sim Pert\left({time}_{trans2RetMin},{time}_{trans2RetMode},{time}_{trans2RetMax}\right)$$

$${temp}_{trans2Ret}\sim Pert\left({temp}_{trans2RetMin},{temp}_{trans2RetMode},{temp}_{trans2RetMax}\right)$$

The caRetRTE() function models the growth of *L. monocytogenes* in RTE diced cantaloupe during retail display, utilizing the caGrowthBaranyi() function to determine growth patterns. This algorithm assumes that all packs of diced cantaloupe from each lot contain bacterial cells with the same physiological state, ln(Qt) passed from the prior logistic phase. All packs from a lot are considered to have the same retail temperature (${temp}_{Ret}$). However, the variation in retail temperatures across different lots is described by a Pert distribution:

$${temp}_{Ret}\sim Pert\left({temp}_{RetMin},{temp}_{RetMode},{temp}_{RetMax}\right)$$

The duration of retail display, however, is determined individually at the unit level. Pert distributions are employed to account for variability in duration for packs on shelves:

$${time}_{Ret}\sim Pert\left({time}_{RetMin},{time}_{RetMode},{time}_{RetMax}\right)$$

The caRet2HomeRTE() function simulates the growth of *L. monocytogenes* in RTE diced cantaloupe during the transportation from retail to home, based on the caGrowthBaranyi() auxiliary function. This algorithm determines transportation time and temperature at the unit level, reflecting variations based on individual consumer behaviors. It uses input data to integrate lot-specific growth rates at 5 °C (EGR_5_) and unit-specific initial quantities of bacteria (ln(Qt)) that are carried over from the preceding logistics stage. The variability in transportation time is modeled using a gamma distribution of parameters defined by [66], while a Pert distribution captures the variability in transportation temperature:

$${time}_{Ret2Home}\sim Gamma\left(scale,{time}_{Ret2HomeMode},{time}_{Ret2HomeMax}\right)$$

Parameter values for the caTrans2RetRTE(), caRetRTE() and caRet2HomeRTE() functions are provided in the Supplementary Materials.

#### 2.1.7. Consumer Handling

**Data and assumptions:**

It is assumed that growth can occur in RTE cantaloupes at home. The variability in temperatures at home may be important and generally further from the target temperature of 4 °C [92]. Rocatto et al. [93] showed that the overall variability in European domestic refrigerators is described by a normal distribution, with a mean of 7 °C for Southern European countries and 6.1 °C for the northern countries. Nauta et al. [66] noted that the average temperature inside European domestic refrigerators was 6.64 °C. A similar value was measured in Brazil [94], with an average refrigerator temperature of 6 °C and with fluctuations ranging from 3.1 to 10.8 °C. Ding et al. [62] used an average refrigerator temperature ranging from 4 to 8.3 °C in a Korean risk assessment model for lettuce. Carrasco et al. [65] estimated the maximum refrigerator temperature at home to be 11.3 °C. RTE diced cantaloupe, a highly perishable food, is expected to be in a consumer’s home for no longer than 10 days [95,96].

**The R function:**

The caHomeRTE() function models the growth of *L. monocytogenes* in RTE diced cantaloupe in home settings, utilizing the caGrowthBaranyi() for underlying growth calculations. This function tailors the simulation of home storage conditions—time and temperature—specifically to each unit, reflecting variations that depend on individual consumer behaviors. It incorporates lot-specific growth rates at 5 °C (EGR_5_) and unit-specific initial bacterial quantities (ln(Qt)) that are derived from earlier stages in the logistics chain. To accurately capture the variability in how consumers store the product at home, Pert distributions are used to model the fluctuations in both storage time and temperature.

$${time}_{Home}\sim Pert\left({time}_{HomeMin},{time}_{HomeMode},{time}_{HomeMax}\right)$$

$${temp}_{Home}\sim Pert\left({temp}_{HomeMin},{temp}_{HomeMode},{temp}_{HomeMax}\right)$$

Parameter values for time and temperature are provided in the Supplementary Materials.

### 2.2. Risk Characterization

Several dose–response relationships are available for *L. monocytogenes* [97]. To estimate the risk of listeriosis per serving, the dose response in the susceptible population of FAO-WHO [98] was chosen. According to this exponential model, each ingested *L. monocytogenes* cell has an independent probability *r* of causing invasive listeriosis, which is assumed to be constant within a given population. The FAO-WHO [98] inferred a median value for *r* of 1.06 × 10^−12^ for the “population with increased susceptibility”. The function DRQuick() from the dose response model R package [97] was employed to calculate the marginal probabilities of invasive listeriosis in the susceptible population *RiskServing_ij_* from the input matrix *N_Cooked ij_*, containing the *L. monocytogenes* doses (CFU) in servings *j* from contaminated lots *i*. The mean risk for every lot *i* (*RiskLot_i_*) was calculated as a risk averaged across servings, *j*:

$${RiskLot}_{i}=\frac{\sum_{j=1}^{c}{RiskServing}_{ij}\times {Prob}_{UnitPos\;i}}{c}$$

### 2.3. QRA Model’s Ouputs

The model’s outputs were summarized at four stages: after harvesting, the end of processing, the point of consumption, and risk characterization. After harvesting, the probability of contamination of lots of cantaloupe and the median contamination are provided. At the end of processing, the descriptors are generated based on the prevalence and the contamination matrix outputs from the function caTesting(). These include the following: (1) descriptive statistics (mean, median, and 95% confidence interval) of the mean concentration of *L. monocytogenes* (CFU/g) in the fraction of contaminated lots; (2) the prevalence of contaminated packs; and (3) the probability that a contaminated pack contains more than 10 CFU *L. monocytogenes* per g of RTE cantaloupe. At the point of consumption, the model’s descriptors are estimated from the outputs of the function caHomeRTE(), which simulates home storage, and include the following: (1) descriptive statistics (mean, median, and 95% confidence interval) of the concentration of *L. monocytogenes* in any serving; (2) the prevalence of contaminated servings; (3) the probability that a contaminated serving contains more than 10 CFU *L. monocytogenes* per g of RTE cantaloupe; and (4) the probability that a contaminated serving contains more than 100 CFU *L. monocytogenes* per g of RTE cantaloupe. For risk characterization, the descriptors include summary statistics such as the mean, median, and 2.5, 97.5, and 99.5 percentiles of the lot-level mean risk per serving, RiskLot.

### 2.4. QRA Model’s Implementation

All the functions described in Section 2.1 were programmed in R version 4.4.1 [99], and compiled in the package qraLm, which can be installed from the Github repository: https://github.com/WorldHealthOrganization/qraLm, accessed on 8 June 2025. The reference manual can be found at https://WorldHealthOrganization.github.io/qraLm/reference/, accessed on 8 June 2025.

## 3. Results and Discussion

### 3.1. Use of the Model for Risk Management Scenarios

**Figure 3:**
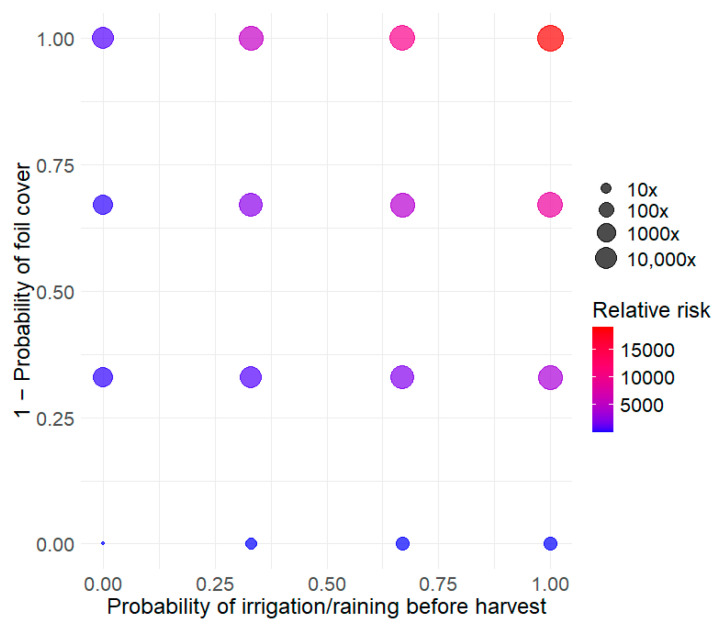
Assessment of the impact of different conditions in the fields on relative listeriosis risk for RTE diced cantaloupe. The reference risk value is defined for all fields using foil (pFoil = 1) and those with an absence of rain or irrigation just before harvesting (pIrrigRaining = 0).

The developed model was used to explore three types of control measures. The first type of intervention concerns the preharvest conditions of cantaloupes. Two parameters were explored: the presence of water on the cantaloupes before harvest (pIrrigRaining) and the use of protective barriers (i.e., barriers (mulch) to prevent contact between the cantaloupes and the soil (pFoil)). Figure 3 shows that these conditions have a strong impact on the risk of listeriosis. Compared to the reference condition (pIrrigRaining = 0, pFoil = 1), not using a barrier and applying overhead irrigation just before harvest across all plots (cantaloupe lots) increases the risk by a factor of 10,000. This increase is not explained by a higher level of contamination at the time of consumption but by a higher prevalence.

**Figure 4:**
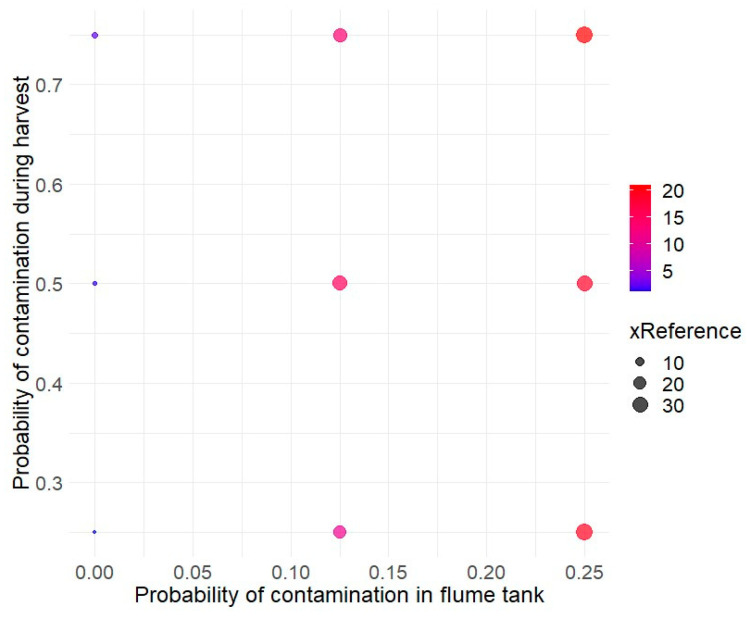
Assessment of the impact of cross-contamination events on relative listeriosis risk for RTE diced cantaloupe. The reference risk value is defined for a probability of cross-contamination during harvest (probCCH) of 0.25 and probability of contamination during washing of cantaloupes (probCCW) of 0.

The hygiene conditions during the harvest and washing of cantaloupes were also explored (Figure 4). The probCCH parameter, which represents hygiene control during harvest, ranged from 0.25 (reference condition) to 0.75, while the probCCH parameter, related to the contamination probability in the washing water, varied from 0 (reference condition) to 0.25. Figure 4 shows that the probability of contamination in the flume tank has a higher impact on the risk of listeriosis than cross-contamination during harvest.

**Figure 5:**
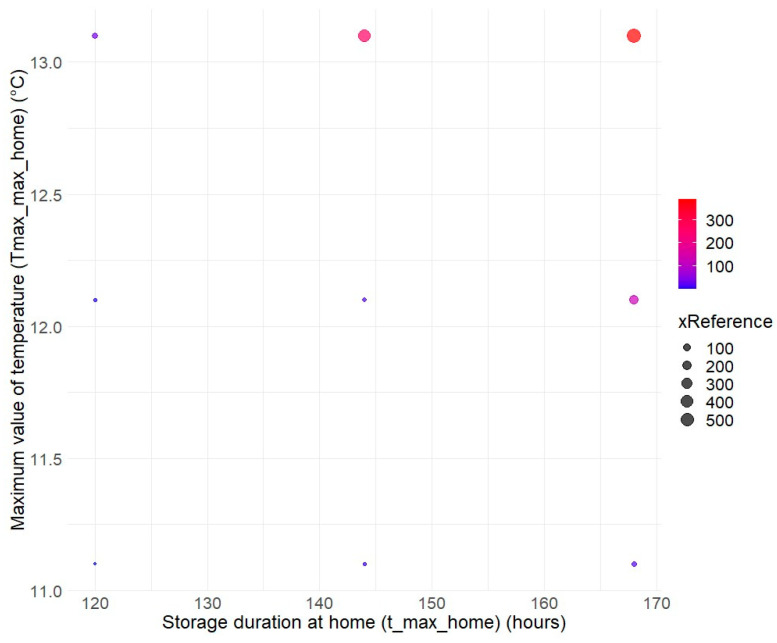
Assessment of the impact of home storage conditions on relative listeriosis risk for RTE diced cantaloupe. The reference risk value is defined for a maximum storage duration at home (*t_max_home*) of 120 h and maximum temperature (*Temp_max_home*) at 11.1 °C.

Finally, as in most risk assessments for listeriosis, the storage conditions at consumers’ homes were explored by varying the maximum values of the triangular distribution for the maximum temperature (Temp_max_home going from 11.1 °C (reference condition) to 13.1 °C) and the maximum storage duration (t_max_home going from 120 h (reference condition) to 168 h). The combination covering the riskiest practices leads to an increase in risk by a factor of 500 (Figure 5).

All parameters used for the reference condition and these different scenarios are listed in the Supplementary Materials presented in the Supplementary Materials.

### 3.2. Validation and Sensitivity Analysis

Johnston et al. [100] investigated the presence of *L. monocytogenes* on melons sampled from 36 fields. None of the samples were positive for the pathogen. Similarly, in our model, under the reference scenario (representing good agricultural practices where all fields use soil barriers and no irrigation occurs just before harvest), the predicted *L. monocytogenes* concentrations per cantaloupe remain below 100 CFU/cantaloupe. However, under the worst-case scenario, where poor agricultural practices are applied, the model predicts that *L. monocytogenes* is present in 73% of cantaloupe batches at the field level. Despite this, given a detection limit of 100 CFU per surface, only 0.05% of individual cantaloupes would test positive. These results highlight the low probability of detecting L. monocytogenes in cantaloupes at the field exit, even when contamination is widespread at the batch level.

Zhang et al. [101] detected *L. monocytogenes* in 0.72% (n = 699) of sampled cut cantaloupes. The model with reference parameters (good agricultural practices and no contamination at the time of washing the cantaloupes) predicts a prevalence of 0.08%. If the contamination frequency is increased to 0.25, then the predicted prevalence is 0.26%.

**Figure 6:**
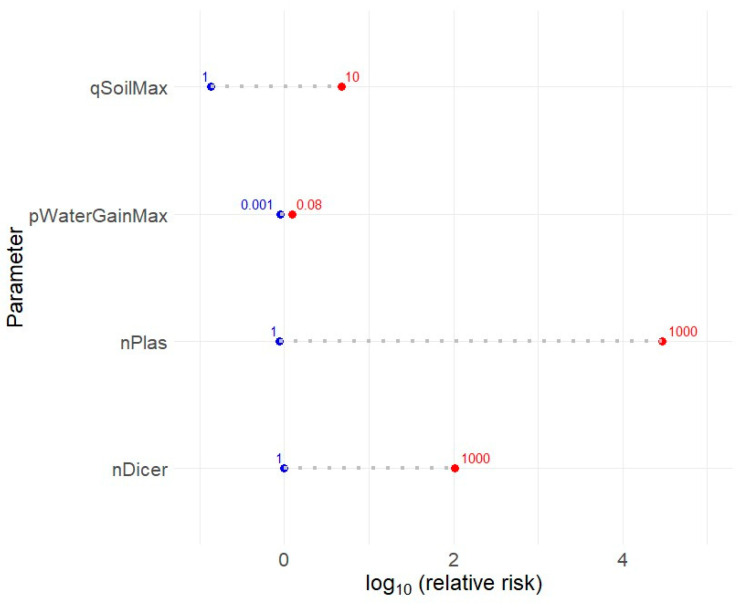
Sensitivity analysis for four parameters of QRA for RTE diced cantaloupe. The values used in the model that provide the reference risk are, respectively, 5g for the max value of the triangular distribution describing variability in the quantity of soil deposited on cantaloupe (qSoilMax), 0.004 for the max value of the % of weight gain (pWaterGainMax), and 9 cfu for the number of *L. monocytogenes* on food contact surfaces (conveyors or crates at harvest) touching the cantaloupes (nPlas) and for the number the surface of the dicing machine (nDicer).

Townsend et al. [16], through a meta-analysis, found that the prevalence of *Listeria* spp. and *L. monocytogenes* on surfaces at the level of harvest or production environment could be very variable, ranging from none to over 50% of samples tested. Although the overall risk may appear low for the model in the reference situation (the mean risk of listeriosis per lot of RTE diced cantaloupe is 3.10^−18^), the risk could be considerably increased by poor hygiene practices at primary production and cantaloupe handling conditions. However, it should be noted that some parameters are poorly supported scientifically and the values used are based on expert elicitation. Figure 6 shows the impact of uncertainty on parameters that appear to have the least evidence. The contamination levels of surface in contact with cantaloupes and present on the dicer appear to have a large effect on risk estimate.

### 3.3. Perspectives and Conclusions

In this study, we evaluated the effectiveness of several relevant “what-if” scenarios using the developed QRA model. This model enables the exploration of other intervention strategies, including the use of higher-quality water for irrigation, assessing the impact of organic fertilizers, reducing contamination during the dicing of RTE cantaloupes, removing highly contaminated lots through targeted sampling plans, shortening shelf life, and exploring combinations of these scenarios, among others.

The recent publication of a dose–response relationship based on the virulence categories of *L. monocytogenes* [97] suggests an improvement in the accuracy of assessments, particularly as initial data are now available on the genomic diversity of *L. monocytogenes* in agricultural soils [102] and production environments [103,104]. An important limitation of the current model is that it does not account for biofilm formation and strain-specific persistence throughout the processing chain. Future modeling efforts incorporating biofilm dynamics and strain tracking could better distinguish the relative contribution of each contamination source and enable more targeted intervention strategies. In conclusion, we have developed a robust set of functions to evaluate the risk of listeriosis associated with the consumption of RTE diced cantaloupe. This QRA has highlighted the potential variations in *L. monocytogenes* risks under different agricultural and processing conditions. The findings from our model underscore the importance of maintaining good agricultural practices in the fields, adhering to rigorous hygiene practices during harvest and processing, and ensuring proper storage conditions by consumers to mitigate the risk of listeriosis. Additionally, the adaptability of our QRA model to different products and processing steps provides significant flexibility for food safety authorities and food business operators around the world, enabling them to tailor interventions specifically to their regional needs.

## Figures and Tables

**Table 5:** Data on the survival of *L. monocytogenes* on intact cantaloupe rind.

Source	Inoculation	Temp. (°C)	Time (Day)	Counts
	Spot-inoculated (6 log_10_) on drawn circles (11 cm^2^) of cantaloupe, then allowed to dry for 1 h.			**log_10_ CFU/cantaloupe (Athena/Rocky Ford Cultivars)**
		4	0	5.77/5.77
			1	4.96/5.57
			3	3.65/4.35
			5	2.78/3.48
			7	2.87/3.39
			9	0.78/2.00
			15	1.75/2.96
		10	0	5.80/5.80
			1	5.59/5.52
			3	4.83/4.35
			5	4.14/4.62
			7	3.73/4.28
			9	2.76/4.00
		25	0	5.85/5.85
			1	5.10/5.10
			3	5.10/2.03
			5	3.30/2.10
			7	3.23/3.23
	Inoculated by immersion in 3 L suspension at 8 log_10_ CFU/mL, then allowed to dry for 1 h.			log_10_ CFU/cm^2^
		4	0	3.47
			1	3.47
			3	3.08
			6	2.93
			9	2.77
			15	2.46
		20	0	3.47
			1	3.47
			3	3.08
			6	2.70
			9	2.31
			15	1.50

**Table 6:** Reduction in *L. monocytogenes* (and standard error) on cantaloupe surface rind after treatment with water alone or with sanitizing agent.

Treatment	Study	Concentration (%)	Exposure Time (min)	Temperature (°C)	Reduction (log_10_ CFU/cm^2^)	St. Error (Reduction)	*n* ^1^
Tap	[76]	-	3	25	0.18	0.053	8
water	[29]		5	25	0.20	-	3
	[77] ^2^						
	Stored at 5 °C x 0 day		5	20	0.20	-	3
	Stored at 5 °C x 7 days		5	20	0.15		3
	[57] ^2^						
	Stored at 4 °C x 1 day	-	2	25	0.30	-	3
	Stored at 4 °C x 5 days	-	2	25	0.22	-	3
	Stored at 4 °C x 15 days	-	2	25	0.22	-	3
ClO_2_ gas	[19]	0.00005	2	25	1.2	-	6
		0.0001	2	25	1.8	-	6
		0.00015	2	25	2.1	-	6
		0.0003	2	25	2.1	-	6
		0.0005	2	25	2.2	-	6
		0.00005	10	25	3.3	-	6
		0.0001	10	25	3.2	-	6
		0.00015	10	25	3.7		6
		0.0003	10	25	3.8	-	6
		0.0005	10	25	4.3	-	6
SH ^3^		0.020	5	25	0.57	0.250	8
							
	Stored at 4 °C x 1 day	0.100	2	25	>3.0	-	3
	Stored at 4 °C x 5 days	0.100	2	25	>3.0	-	3
	Stored at 4 °C x 15 days	0.100	2	25	>3.0	-	3
H_2_O_2_	[29]	2.5	5	25	2.8		3
	[77] ^2^						
	Stored at 5 °C x 0 day	2.5	5	20	2.3	-	3
	Stored at 5 °C x 7 days	2.5	5	20	2.8		3
	[57] ^2^						
	Stored at 4 °C x 1 day	5.0	2	25	>3.0	-	3
	Stored at 4 °C x 5 days	5.0	2	25	>3.0	-	3
	Stored at 4 °C x 15 days	5.0	2	25	>3.0	-	3
HPLNC ^4^	[77] ^2^						
	Stored at 5 °C x 0 day	-	5	20	>4.0	-	3
	Stored at 5 °C x 7 days	-	5	20	>4.0	-	3

^1^ Number of samples in inoculation experiments. ^2^ Studies where inoculated cantaloupes were stored at different temperatures and times before the washing treatment. ^3^ Sodium hypochlorite. ^4^ Solution of 1% H_2_O_2_, 25 μg/mL nisin, 1% sodium lactate and 0.5% citric acid.

**Table 7:** Available data to build a transfer equation of *L. monocytogenes* load from cantaloupe rind to flesh (diced pieces in g or surface slices in cm^2^).

Source		*L. monocytogenes* on the Rind (log_10_ CFU/cm^2^)	*L. monocytogenes* in Cantaloupe Flesh	Transfer Rate (%) (10^flesh^/10^rind^) × 100
To fresh-cut (diced pieces)			(log_10_ CFU/g)	
[20]		2.16	0.23	1.175
		3.26	0.54	0.191
		3.98	1.31	0.214
		4.52	1.46	0.087
[29]		4.60	2.60	1.000
		4.40	2.20	0.631
To slices (flesh surface)			(log_10_ CFU/cm^2^)	
[81]	4 °C	5.94	2.45	0.032
		5.44	1.42	0.010
	30 °C	5.22	1.64	0.026
		5.44	1.17	0.005

**Table 8:** Growth kinetics data of *L. monocytogenes* in cantaloupe flesh or similar from published articles.

Study	Medium	Strain	Stressed	Temperature (°C)	Specific GR (h^−1^)
[61]	Cantaloupe	F2365, H7858, ATCC19115	Stressed	4.0	0.0120
			(rifampicin-resistant and cold-resistant)	8.0	0.0470
				12	0.1260
				16	0.1860
				20	0.2930
				25	0.5250
				30	0.7300
				33	0.8150
				40	0.9160
				37	0.8600
				43	0.6920
		F4260	Stressed (rifampicin-resistant and cold-resistant)	4.0	0.0110
				8.0	0.0580
				12	0.1230
				16	0.1940
				20	0.3210
				25	0.5300
				30	0.7470
				33	0.9000
				37	0.9900
				40	0.9730
				43	0.7590
				38	0.9750
		V7	Stressed (rifampicin-resistant and cold-resistant)	43	0.7540
				40	0.9980
				33	0.9010
				37	0.9640
				30	0.7860
				25	0.5430
				20	0.3220
				16	0.1970
				12	0.1350
				8.0	0.0640
[87]	Cantaloupe	ATCC BAA839, ATCC BAA839, ATCC 19111, ATCC 13932	Stressed (rifampicin-resistant and cold-resistant)	10	0.0852
				15	0.1983
				20	0.4030
				25	0.5803
				10	0.0852
				15	0.2118
				20	0.4030
				25	0.5112
	Diced cantaloupe	Scott A, H7778, ATCC-15313, CCR1LG	Not stressed	20	0.3720
	Fresh-cut cantaloupe	LCDC 81-861, Scott A, 101M, V7	Not stressed	5.0	0.0850
				10	0.2000
				15	0.2300
				20	0.3900
				25	0.4745
	Fresh-cut cantaloupe	NRCC B33076	Not stressed	5.0	0.0368
				10	0.0898
				25	0.6240
				30	0.7161
				36	0.9233
[81]	Diced cantaloupe	J22F, J29H, M3	Not stressed	4.0	0.0520
				7.0	0.0670
				10	0.1840
[88]	Fresh-cut watermelon	LCDC 81-861, V7, 101M, Scott A	Not stressed	4.0	0.0318
				13	0.1213
				13	0.1438
[86]	Squash	Not stated	Not stressed	4.0	0.0370
				10	0.0910

## Data Availability

The data presented in this study are openly available in [https://github.com/WorldHealthOrganization/qraLm, accessed on 8 June 2025]. The data employed to build the quantitative risk assessment model are published elsewhere, and can be directly extracted from the articles cited.

## Supplementary Materials

The following supporting information can be downloaded at https://www.mdpi.com/article/10.3390/foods14132212/s1: Table of parameters allowing us to define temperatures and duration for the transport module.
